# Defining genetic diversity of rhesus macaque Fcγ receptors with long-read RNA sequencing

**DOI:** 10.3389/fimmu.2023.1306292

**Published:** 2024-01-09

**Authors:** Haleigh E. Conley, Max M. He, David Easterhoff, Hélène Fradin Kirshner, Sarah L. Cocklin, Jacob Meyer, Taylor Hoxie, Madison Berry, Todd Bradley, William D. Tolbert, Marzena Pazgier, Georgia D. Tomaras, Joern E. Schmitz, Michael Anthony Moody, Kevin Wiehe, Justin Pollara

**Affiliations:** ^1^ Department of Surgery, Duke University School of Medicine, Duke University, Durham, NC, United States; ^2^ Duke Human Vaccine Institute, Duke University School of Medicine, Duke University, Durham, NC, United States; ^3^ Center for Virology and Vaccine Research, Beth Israel Deaconess Medical Center, Harvard Medical School, Boston, MA, United States; ^4^ Genomic Medicine Center, Children’s Mercy Kansas City, Kansas City, MO, United States; ^5^ Infectious Disease Division, Department of Medicine, Uniformed Services University of the Health Sciences, Bethesda, MD, United States

**Keywords:** rhesus macaques, Fc receptor, Long-read RNA sequencing, FcγR SNPs, FcγR structures, genetic diversity

## Abstract

Fcγ receptors (FcγRs) are membrane-bound glycoproteins that bind to the fragment crystallizable (Fc) constant regions of IgG antibodies. Interactions between IgG immune complexes and FcγRs can initiate signal transduction that mediates important components of the immune response including activation of immune cells for clearance of opsonized pathogens or infected host cells. In humans, many studies have identified associations between FcγR gene polymorphisms and risk of infection, or progression of disease, suggesting a gene-level impact on FcγR-dependent immune responses. Rhesus macaques are an important translational model for most human health interventions, yet little is known about the breadth of rhesus macaque FcγR genetic diversity. This lack of knowledge prevents evaluation of the impact of FcγR polymorphisms on outcomes of preclinical studies performed in rhesus macaques. In this study we used long-read RNA sequencing to define the genetic diversity of FcγRs in 206 Indian-origin Rhesus macaques, *Macaca mulatta*. We describe the frequency of single nucleotide polymorphisms, insertions, deletions, frame-shift mutations, and isoforms. We also index the identified diversity using predicted and known rhesus macaque FcγR and Fc-FcγR structures. Future studies that define the functional significance of this genetic diversity will facilitate a better understanding of the correlation between human and macaque FcγR biology that is needed for effective translation of studies with antibody-mediated outcomes performed in rhesus macaques.

## 1 Introduction

Fragment crystallizable receptors (FcRs) are antibody isotype-specific binding molecules mainly expressed by myeloid and lymphoid cells that play a fundamental role in regulating antibody mediated effector functions and cell activation (1). The Type I receptors for IgG — Fcγ receptors (FcγRs) — are clinically important as IgG Fc–FcγR interactions initiate a variety of immune functions including antibody-dependent cellular cytotoxicity (ADCC), antibody-dependent cellular phagocytosis (ADCP), immune complex-mediated antigen presentation, and enhancement of antibody neutralization activity (2–7).

Humans express six extracellular type I Fc receptors that interact with the IgG Fc domain. Cell activation is tightly regulated by complex interactions with either activating receptors FcγRI, FcγRIIa, FcγRIIc, FcγRIIIa and FcγRIIIb or the inhibitory receptor FcγRIIb (1, 6). Allelic variation of human FcγR includes single nucleotide polymorphisms (SNPs) impacting amino acids located within IgG contact regions. These SNPs are associated with predisposition to infections (8–10), autoimmunity (9, 11, 12), outcomes of solid organ transplantation (13, 14), efficacy of immunotherapies (15, 16), and decreased risk of HIV-1 acquisition in HIV-1 vaccine trials (17–22). Hence, FcγR genetic variation can have broad impacts on human health, including outcomes of health interventions.

Rhesus macaques are an essential preclinical research model for understanding the etiology of disease (23) and for developing new biomedical interventions and therapies, such as vaccines and antibody-based biologic drugs (24). Considering the important roles of FcγRs and antibody-mediated effector functions identified in human studies and the numerous identified impacts of FcγR genetic variation on health outcomes, it is clear that a comprehensive characterization of rhesus macaque FcγR genetic variation is critical for interpreting the translatability of disease progression, passive antibody administration, and active vaccination studies performed in rhesus macaques.

Rhesus macaques are anatomically, physiologically, and phylogenetically similar to humans. Rhesus macaques and humans have approximately 93% overall genomic sequence identity (25), and homologous type I FcγR genes have been identified in rhesus macaques. Rhesus FcγRI has 94%-95% sequence similarity to human FcγRIa (26), FcγRIIA has 88%-90% sequence similarity to human FcγRIIa (26), FcγRIIB has 87% sequence similarity to human FcγRIIb (27), and FcγRIII has 91.7% sequence similarity to human FcγRIIIa (28). Prior investigations of genetic diversity in rhesus macaque type I FcγR genes have been limited in either the number of genes evaluated, the number or source of rhesus macaques, or the sequencing technology utilized (26, 28, 29). Our study evaluated FcγR genetic variation of rhesus macaques from at least 5 study sites. We also identified isoforms of FcγRIIA, FcγRIIB, and FcγRIII.

The purpose of this study was to perform a comprehensive analysis of Indian origin rhesus macaque type I FcγR sequences to identify the magnitude of genetic variation. We used Pacific Biosciences (PacBio) long-read sequencing technology to identify diversity of actively transcribed type I FcγR genes from peripheral blood mononuclear cells (PBMCs) isolated from 206 rhesus macaques. To date, this is the largest survey of rhesus macaque FcγR genetic diversity. We report the frequency of single nucleotide polymorphisms (SNPs), insertions, deletions, frame-shift mutations, and isoforms. We also used AlphaFold2 structural predictions (30) to map the locations of genetic variability onto models of FcγR structures to provide insight on the possible implications of nonsynonymous mutations. Our approach and data form an organized framework for systematically cataloging rhesus macaque type I FcγR genetic variation to identify functionally significant FcγR polymorphisms that may influence study outcomes and translation.

## 2 Methods

### 2.1 Peripheral blood collection

Peripheral blood was collected by venipuncture from adult Indian origin rhesus macaques (Macaca mulatta) enrolled in a diverse assortment of research studies at multiple institutions. Specifically, leftover blood samples from completed studies were used for the current study. These blood samples represent 206 Indian origin rhesus macaques, sourced from several different vendors, providers, or colonies. All blood was collected in accordance with protocols approved by the appropriate Institutional Animal Care and Use Committees.

### 2.2 Reverse transcription-PCR amplification of FcγR genes

Cryopreserved rhesus macaque (RM) PBMCs were rapidly thawed and washed with RPMI media (Gibco, Thermo Fisher Scientific, Waltham, MA) supplemented with 10% fetal bovine serum (FBS). Cell count and viability was obtained with a Muse^®^ Count and Viability Assay (Luminex Corporation, Austin, TX). RNA and genomic DNA (gDNA) was extracted using AllPrep DNA/RNA isolation kit (Qiagen, Germantown, MD) following the manufacturer’s protocol. Total RNA was reverse transcribed in a 20 µL reaction volume with the QuantiTect Reverse Transcription Kit (Qiagen). First, potential contaminating gDNA was eliminated by adding 2µL of gDNA Wipeout Buffer to ≤1µg of RNA in DNase/RNase free water (Gibco, total reaction volume of 14 µL) and incubating for 2 minutes at 42°C. Then 1µL of Quantiscript reverse transcriptase, 4µL Quantiscript RT buffer and 1µL of RT primer mix was added. Reverse transcription reaction was performed at 42°C for 15 minutes and then 95°C for 3 minutes. RM FcγR genes were then amplified by PCR using gene-specific primers designed with PacBio barcodes (Pacific Biosciences, Menlo Park, CA, **Figure S1**). All PCR reactions were performed in 25µL reaction volume containing 3µL cDNA, 0.5µL 10µM Forward primer, 0.5µL 10µM Reverse primer, 12.5µL Platinum Hot Start Master Mix (Thermo Fisher) and 8.5µL DNase/RNase free water (Gibco). FcγR gene amplification was performed at 94°C for 2 minutes, 94°C 30 seconds, 64°C 30 seconds, 72°C for 1 minute, 72°C for 7 minutes with 34 cycles. PCR products were purified using ZR-96 DNA clean and Concentrator™-5 (Zymo Research, Irvine, CA) following the manufacturer’s protocol. Samples were eluted in 20µL volume of elution buffer.

### 2.3 Long-read RNA sequencing

PacBio SMRTbell library preparation was performed in accordance with manufacturer’s recommendations (Pacific Biosciences, Menlo Park, CA). DNA concentrations were determined by Qbit quantification (Thermo Fisher) and equal concentrations of ~30 amplicons were pooled and loaded onto a single SMRT cell. Sequencing was performed on a PacBio Sequel instrument using version 2.1 chemistry (Pacific Biosciences).

### 2.4 PacBio sequence analysis and variant identification

The subreads of PacBio datasets were demultiplexed using PacBio Lima (version 2.0.0) (https://github.com/pacificbiosciences/barcoding/) and CCS (version 6.00) (https://github.com/PacificBiosciences/ccs) for generating HiFi circular consensus sequence (CCS) reads. Quality controls were assessed by analyzing productivity and sequence read length before and after trimming. Data analysis was conducted using a pipeline similar to that previously described (31). HiFi CCS reads were aligned to the latest version reference sequences of rhesus macaque rheMac10 (http://hgdownload.soe.ucsc.edu/goldenPath/rheMac10/bigZips/) using the long-read sequence alignment tool minimap2 (version 2.17) (32). SNPs and small insertions/deletions (indels) were identified using the GATK Haplotype Caller (version 4.1.9) using the default parameters recommended by GATK for long-read variant calling including a MAPQ threshold of >50 (33). Variants were then annotated using the program ANNOVAR (34). We retained only variants that were detected in at least 2% of the animals within the cohort.

### 2.5 Isoform analysis

The relative abundances of isoform-specific gene expression were estimated by using long-read RNA-seq data aligned to reference isoforms of rhesus macaque FcγR genes using LIQA (35). To obtain reference isoforms, we retrieved all rhesus macaque isoforms from the rheMac10 assembly via the UCSC genome browser (http://genome.ucsc.edu) and subsequently selected the subset specific to the FcγR isoforms. For downstream analysis and visualization of isoforms, we applied a selection criterion. Specifically, we included only those isoforms that showed a relative abundance greater than 1% in at least one monkey for each FcγR gene. Identification of novel isoforms was not attainable due to limitations of the Pacbio sequencing protocol that was performed.

### 2.6 Whole genome sequencing and FcγR gDNA analysis

Purified gDNA (isolated as described above) was prepped using the Nextera DNA Library Prep Kit (Illumina, San Diego, CA) and sequenced using the Illumina 150 bp NextSeq 6000 platform.

Both whole genome sequencing data and FcγR gDNA short-read data were aligned to the reference rheMac10 genome using BWA version 0.7.17 (36). Variants including both SNPs and indels were identified using the GATK Haplotype Caller (version 4.1.9) using the default parameters for short-read sequencing (33) and annotated with ANNOVAR (34).

### 2.7 AlphaFold2 structure predictions

Detailed atomic-level models of protein structures were predicted using AlphaFold2 (https://github.com/deepmind/alphafold/) (30) with default parameters. All models shown are the top scoring model from AlphaFold2.

### 2.8 Receptor – IgG interface predictions

Nonsynonymous SNPs were mapped onto the crystal structures of human FcγR receptor complexes with human IgG1 Fc (FcγRI, FcγRIIa, and FcγRIIb) and the macaque FcγRIII complex with macaque IgG1 Fc available in the Brookhaven Protein Databank (PDB IDs 4ZNE, 3RY6, 3WJJ, and 7KCZ respectively). FcγRI Fc contact residues include 85-88, 109-111, 113-117, 119, 124-129, 131-132, and 155-159 based upon human FcγRI-Fc complex structures PDB IDs 4w40 and 4zne. FcγRIIa Fc contact residues include: 86-87, 110, 114, 116-117, 125-127, 129, 131, and 155-157 based upon the human FcγRIIa-Fc complex structure PDB ID 3RY6. FcγRIIb Fc contact residues: 85-87, 110, 113, 117, 119, 126-131, and 157-160 based upon the human FcγRIIb-Fc complex structure PDB ID 3WJJ. Figures were made with Pymol (http://pymol.org).

## 3 Results

### 3.1 FcγR primer design

Most rhesus macaque FcγR type I primers have been designed to regions of homology with human FcγR type I genes (26, 28). Use of similar designed primers in this study identified several rhesus macaques refractory to RT-PCR amplification. Since very few rhesus macaque FcγR type I reference sequences were publicly available to refine primer design, two rhesus macaques refractory to RT-PCR amplification were whole genome shotgun sequenced on the Illumina platform. FcγR type I sequences were aligned to Mmul_8.0.1 and new rhesus macaque FcγR type I gene specific primers were designed to the 5′ and 3′ untranslated region of each FcγR gene. In addition, primer design was cross-checked with publicly available rhesus macaque genome sequencing data (37). To increase coverage for FcγRIII, two forward primers were used (**Figure S1A**).

Primer design was validated using RNA extracted from cryopreserved rhesus macaque PBMCs. FcγR genes were RT-PCR amplified and assessed by gel electrophoresis (**Figures S1B, C**). Visible bands were gel extracted and Sanger sequenced. All primers efficiently amplified FcγR genes with the expected molecular weight (Gene + untranslated region) FcγRI ~1000 bp; FcγRIIa, FcγRIIb, FcγRIIIa ~800bp (26) (**Figure S1C**) and were confirmed by sequencing to be the indicated FcγR gene.

### 3.2 FcγR polymorphisms

To probe rhesus macaque FcγR sequence diversity, FcγRI, FcγRIIa, FcγRIIb and FcγRIII genes were RT-PCR amplified from 206 rhesus macaques using the newly designed primers with symmetric PacBio barcodes added. PCR amplicons underwent long-read sequencing on a PacBio Sequel instrument as described in the Methods section. FcγR sequences were then aligned to references sequences from the rheMac10 draft genome.

**Figure 1:**
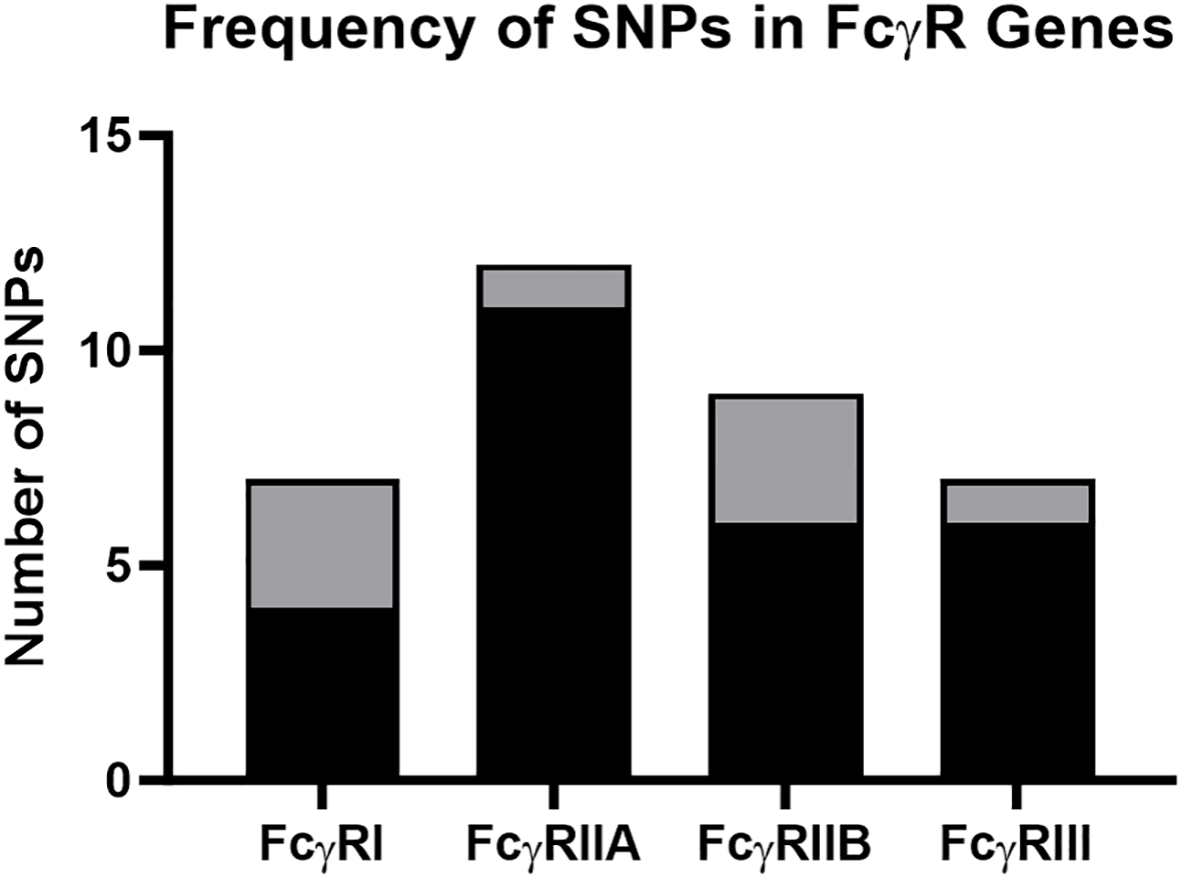
Frequency of SNPs detected in rhesus macaque FcγR genes. The total number of nonsynonymous and synonymous SNPs for FcγRI, FcγRIIa, FcγRIIb and FcγRIII. Black shading indicates previously described SNPs, and gray shading indicates previously undescribed SNPs identified in our study.

We detected multiple SNPs in rhesus macaque FcγRI, FcγRIIa, FcγRIIb and FcγRIII genes (**Figure 1**). We have summarized the observed genetic variation in **Tables 1**–**4**. In these tables, we first indicate the chromosomal location of the variant. We also provide a description of the type of genetic variation: nonsynonymous SNPs, synonymous SNPs, or insertions (frame shift or non-frame shift). We next provide the nucleotide mutation and amino acid changes based on ANNOVAR numbering relative to the rheMac10 draft genome (34). Position 1 in ANNOVAR numbering is where the open reading frame begins. We also included nucleotide and amino acid numbering based on the mature protein: Position 1 is the first amino acid of the predicted mature protein sequence with the signal peptide amino acids are denoted by negative integers. We refer to the mature protein numbering throughout the manuscript. Similarly, all isoform descriptions are based on the mature protein amino acid positions. Finally, the tables also indicate the percentage of rhesus macaque carriers for each variant among the 206 animals sequenced. SNP detection with PacBio was confirmd with Sanger sequencing (**Table S1**).

**Table 1:** FcγRI genetic variation observed in 206 Indian-origin rhesus macaques using long-read RNA sequencing.

Variant	Description	Exon	ANNOVAR NT^1^	ANNOVAR AA^2^	Transcript NT^3^	Mature protein AA^4^	Domain	% Carriers^5^
chr1:101378924:A>G	nonsynonymous SNP^6^	3	c.T134C	p.V45A	T83C	V28A	Extracellular	25.2 (52/206)
chr1:101378806:C>G	nonsynonymous SNP	3	c.G252C	p.R84S	G201C	R67S	Extracellular	17.5 (36/206)
chr1:101378828:A>G	nonsynonymous SNP	3	c.T230C	p.V77A	T179C	V60A	Extracellular	13.6 (28/206)
chr1:101378934:G>C	nonsynonymous SNP	3	c.C124G	p.Q42E	C73G	**Q25E**	Extracellular	4.8 (10/206)
chr1:101374528:G>A	nonsynonymous SNP	4	c.C457T	p.L153F	C406T	**L136F**	Extracellular	3.4 (7/206)
chr1:101378991:>T	fs^7^ insertion	3	c.66dupA^8^	p.V23fs	15dupA	V6fs	Extracellular	2.9 (6/206)
chr1:101373136:C>T	synonymous SNP	5	c.G603A	p.P201P	G552A	P184P	Extracellular	2.4 (5/206)
chr1:101378972:G>A	nonsynonymous SNP	3	c.C86T	p.P29L	C35T	**P12L**	Extracellular	2.4 (5/206)

Only variants found in more than 2% of sequenced animals are shown.

^1^Nucleotide mutation by ANNOVAR numbering relative to NCBI reference sequence NM_001257304, ^2^Amino acid mutation by ANNOVAR numbering, ^3^Nucleotide mutation using full transcript numbering, ^4^Amino acid mutation by mature protein numbering, ^5^percentage of carriers from 206 sequenced animals, ^6^SNP: Single nucleotide polymorphism, ^7^fs: Frame shift, ^8^dup: duplication. All mutations are shown in relation to the reference sequences from the rheMac10 draft genome. Bolded text in mature protein AA column indicates nonsynonymous SNP not previously described in the literature.

**Table 2:** FcγRIIA genetic variation observed in 206 Indian-origin rhesus macaques using long-read RNA sequencing.

Variant	Description	Exon	ANNOVAR NT^1^	ANNOVAR AA^2^	Transcript NT^3^	Mature protein AA^4^	Domain	% Carriers^5^
chr1:89419258:>ATC	non-fs^6^ insertion	7	c.894_895insGAT^7^	p.R299delinsDR^8^	789_790insGAT	R264delinsDR	Cytoplasmic	68 (140/206)
chr1:89431476:C>T	synonymous SNP	3	c.G114A	p.P38P	G9A	P3P	Extracellular	11.6 (24/206)
chr1:89431321:A>C	nonsynonymous SNP	3	c.T269G	p.M90R	T164G	M55R	Extracellular	11.6 (24/206)
chr1:89431407:A>G	synonymous SNP	3	c.T183C	p.C61C	T78C	C26C	Extracellular	11.1 (23/206)
chr1:89427299:G>T	nonsynonymous SNP	4	c.C489A	p.N163K	C384A	N128K	Extracellular	11.1 (23/206)
chr1:89431374:G>A	synonymous SNP	3	c.C216T	p.T72T	C111T	T37T	Extracellular	11.1 (23/206)
chr1:89419246:T>G	nonsynonymous SNP	7	c.A907C	p.M303L	A802C	**M268L**	Cytoplasmic	10.7 (22/206)
chr1:89427309:T>A	nonsynonymous SNP	4	c.A479T	p.K160I	A374T	K125I	Extracellular	7.8 (16/206)
chr1:89427264:T>C	nonsynonymous SNP	4	c.A524G	p.Q175R	A419G	Q140R	Extracellular	6.3 (13/206)
chr1:89427254:G>A	synonymous SNP	4	c.C534T	p.H178H	C429T	H143H	Extracellular	3.9 (8/206)
chr1:89427291:T>G	nonsynonymous SNP	4	c.A497C	p.H166P	A392C	H131P	Extracellular	2.4 (5/206)
chr1:89427286:T>C	nonsynonymous SNP	4	c.A502G	p.N168D	A397G	N133D	Extracellular	2.4 (5/206)
chr1:89419298:G>A	synonymous SNP	7	c.C855T	p.G285G	C750T	G250G	Cytoplasmic	2.4 (5/206)

Only variants found in more than 2% of sequenced animals are shown.

^1^Nucleotide mutation by ANNOVAR numbering relative to NCBI reference sequence NM_001257300, ^2^Amino acid mutation by ANNOVAR numbering, ^3^Nucleotide mutation using full transcript numbering, ^4^Amino acid mutation by mature protein numbering, ^5^percentage of carriers from 206 sequenced animals, ^6^fs: Frame shift, ^7^ins: Insertion, ^8^delins: Deletion/Insertion, ^9^SNP: Single nucleotide polymorphism. All mutations are shown in relation to the reference sequences from the rheMac10 draft genome. Bolded text in mature protein AA column indicates nonsynonymous SNP not previously described in the literature.

**Table 3:** FcγRIIB genetic variation observed in 206 Indian-origin rhesus macaques using long-read RNA sequencing.

Variant	Description	Exon	ANNOVAR NT^1^	ANNOVAR AA^2^	Transcript NT^3^	Mature protein AA^4^	Domain	% Carriers^5^
chr1:89331119:T>C	nonsynonymous SNP	4	c.A532G	p.N178D	A397G	N133D	Extracellular	20.9 (43/206)
chr1:89331140:A>C	nonsynonymous SNP	4	c.T511G	p.S171A	T376G	**S126A**	Extracellular	14.6 (30/206)
chr1:89331111:G>A	synonymous SNP	4	c.C540T	p.N180N	C405T	N135N	Extracellular	14.1 (29/206)
chr1:89326856:A>G	synonymous SNP	7	c.T846C	p.D282D	T711C	D237D	Extracellular	8.2 (17/206)
chr1:89331097:T>C	nonsynonymous SNP	4	c.A554G	p.Q185R	A419G	**Q140R**	Extracellular	5.8 (12/206)
chr1:89331161:T>C	nonsynonymous SNP	4	c.A490G	p.T164A	A355G	**T119A**	Extracellular	4.4 (9/206)
chr1:89331142:A>T	nonsynonymous SNP	4	c.T509A	p.I170K	T374A	I125K	Extracellular	2.9 (6/206)
chr1:89331246:G>A	synonymous SNP	4	c.C405T	p.L135L	C270T	L90L	Extracellular	2.9 (6/206)
chr1:89331024:A>G	synonymous SNP	4	c.T627C	p.P209P	T492C	P164P	Extracellular	2.4 (5/206)

Only variants found in more than 2% of sequenced animals are shown.

^1^Nucleotide mutation by ANNOVAR numbering relative to NCBI reference sequence NM_001257302, ^2^Amino acid mutation by ANNOVAR numbering, ^3^Nucleotide mutation using full transcript numbering, ^4^Amino acid mutation by mature protein numbering, ^5^percentage of carriers from 206 sequenced animals, ^6^SNP: Single nucleotide polymorphism. All mutations are shown in relation to the reference sequences from the rheMac10 draft genome. Bolded text in mature protein AA column indicates nonsynonymous SNP not previously described in the literature.

**Table 4:** FcγRIII genetic variation observed in 206 Indian-origin rhesus macaques using long-read RNA sequencing.

Variant	Description	Exon	ANNOVAR NT^1^	ANNOVAR AA^2^	Transcript NT^3^	Mature protein AA^4^	Domain	% Carriers^5^
chr1:89392794:G>A	nonsynonymous SNP^6^	5	c.G805A	p.V269I	G643A	V215I	Cytoplasmic	67.5 (139/206)
chr1:89392782:G>A	nonsynonymous SNP	5	c.G793A	p.V265M	G631A	V211M	Cytoplasmic	67 (138/206)
chr1:89387252:G>A	synonymous SNP	3	c.G411A	p.Q137Q	G249A	Q83Q	Extracellular	17 (35/206)
chr1:89390827:A>G	nonsynonymous SNP	4	c.A634G	p.I212V	A472G	I158V	Extracellular	7.8 (16/206)
chr1:89392727:T>C	synonymous SNP	5	c.T738C	p.S246S	T576C	S192S	Extracellular	3.9 (8/206)
chr1:89390721:G>A	synonymous SNP	4	c.G528A	p.T176T	G366A	T122T	Extracellular	3.9 (8/206)
chr1:89387025:G>T	nonsynonymous SNP	3	c.G184T	p.A61S	G22T	**A8S**	Extracellular	2.9 (6/206)

Only variants found in more than 2% of sequenced animals are shown.

^1^Nucleotide mutation by ANNOVAR numbering relative to NCBI reference sequence NM_001271653 where position 1 starts with the open reading frame (position 54 in reference NM_001271653.1_RhM), ^2^Amino acid mutation by ANNOVAR numbering, ^3^Nucleotide mutation using full transcript numbering, ^4^Amino acid mutation by mature protein numbering, ^5^percentage of carriers from 206 sequenced animals, ^6^SNP: Single nucleotide polymorphism. All mutations are shown in relation to the reference sequences from the rheMac10 draft genome. Bolded text in mature protein AA column indicates nonsynonymous SNP not previously described in the literature.

**Figure 2:**
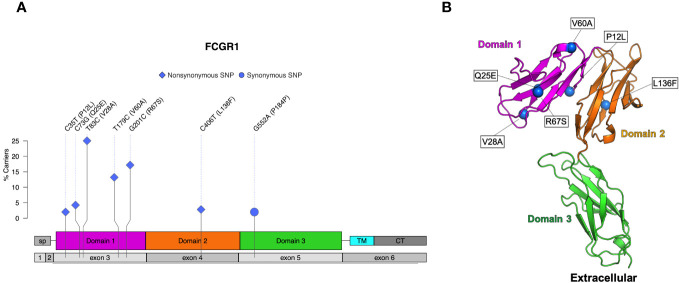
Identified SNPs in FcγRI. **(A)** Lollipop diagram showing frequency, domain location, and type of observed SNP. **(B)** AlphaFold2 plot showing the extracellular domain and the corresponding amino acid changes (magenta = domain 1, orange = domain 2, cyan = transmembrane, gray = other regions).

Six nonsynonymous and one synonymous FcγRI SNPs were identified in more than 2% of the 206 sequenced animals: V28A, R67S, V60A, Q25E, L136F, P12L (**Table 1**; **Figure 2A**). We used AlphaFold2 modeling (30) to predict the mature protein structures and map the location of the amino acid changes associated with the nonsynonymous SNPs (**Figure 2B**). V28A, R67S, V60A, and Q25E all occur in the extracellular domains. They are also outside of the predicted Fc binding region of FcγRI (**Figure S2A**) based upon human FcγRI-Fc complex structures (PDB IBDs: 4W40 and 4ZNE).

**Figure 3:**
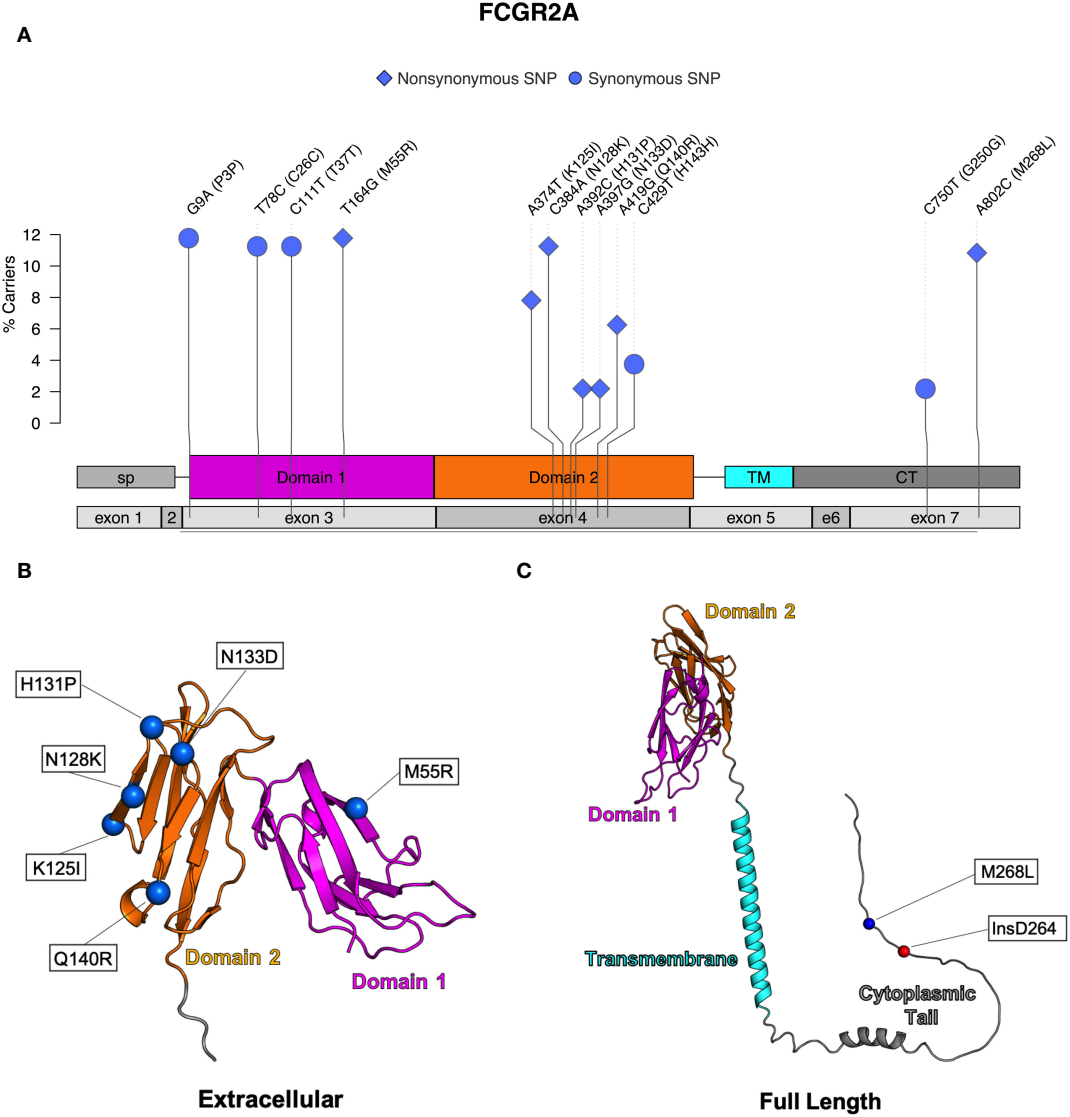
Identified SNPs in FcγRIIa. **(A)** Lollipop diagram showing frequency, domain location, and type of observed SNP. AlphaFold2 plots showing corresponding amino acid changes in the **(B)** extracellular domain and **(C)** the cytoplasmic domain (magenta = domain 1, orange = domain 2, cyan = transmembrane, gray = other regions).

Seven nonsynonymous and five synonymous SNPs were identified in FcγRIIa (**Table 2**; **Figure 3A**). As shown in **Figures 3B, C**, AlphaFold2 predictions indicate that all but one nonsynonymous SNPs occur in the extracellular domain. M268L occurs in the cytoplasmic tail of FcγRIIa. One SNP, N128K, occurs at an N-linked glycosylation site and results in the elimination of the glycan site. Four of the extracellular nonsynonymous SNPs occur in residues outside the predicted FcγRIIa-Fc binding interface (M55R, N128K, N133D, and Q140R). Two SNPs occur within the predicted FcγRIIa-Fc interface based upon the human FcγRIIa-Fc complex structure (PDB ID: 3RY6) (K125I and H131P) (**Figure S2B**). Due to its proximity to binding residues and removal of a glycosylation site, the N128K SNP also likely modulates Fc binding; however, it is unclear from the structure if the N133D and Q140R SNPs are close enough to the interface to have an impact.

**Figure 4:**
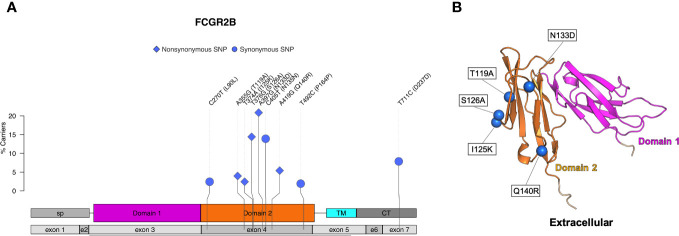
Identified SNPs in FcγRIIb. **(A)** Lollipop diagram showing frequency, domain location, and type of observed SNP. **(B)** AlphaFold2 plot showing the extracellular domain and the corresponding amino acid changes (magenta = domain 1, orange = domain 2, cyan = transmembrane, gray = other regions).

Five nonsynonymous SNPs were identified in FcγRIIb: T119A, I125K, S126A, N133D, and Q140R along with four synonymous SNPs (**Table 3**; **Figure 4A**). As shown in **Figure 4B**, AlphaFold2 predicts all SNPs occur in the extracellular domain of FcγRIIb. Two of the nonsynonymous SNPs (T119A and S126A) occur in residues within the predicted FcγRIIb-Fc interface (based upon the human FcγRIIb-Fc complex structure PDB ID 3WJJ), and two are adjacent to the predicted interface (I125K and N133D) (**Figure S2C**) It is unclear from the structure if the Q140R SNP is close enough to the interface to have an impact on Fc binding.

**Figure 5:**
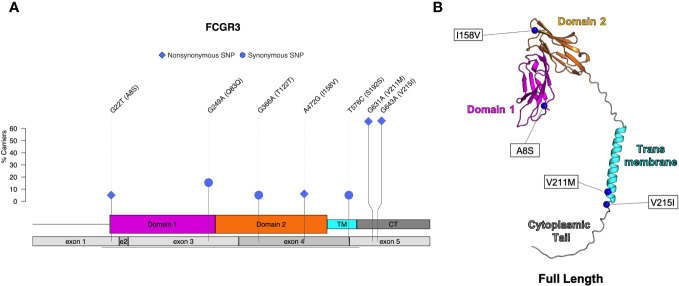
Identified SNPs in FcγRIII. **(A)** Lollipop diagram showing frequency, domain location, and type of observed SNP. **(B)** AlphaFold2 plot of full-length protein with corresponding amino acid changes (magenta = domain 1, orange = domain 2, cyan = transmembrane, gray = other regions).

Four nonsynonymous SNPs were identified in FcγRIII: V215I, V211M, I158V, and A8S (**Table 4**; **Figure 5A**). We also identified three synonymous SNPs. AlphaFold2 predictions indicate that SNPs V215I and V211M occur in the cytoplasmic tail, and I158V and A8S occur in the extracellular domain (**Figure 5B**) (29, 38). The I158V SNP occurs at the same Fc-FcR contact region (**Figure S2D**) as the human FcγRIIIa V158F. The V158F SNP reduces binding to human IgG1 and abrogates binding to human IgG2 and IgG4 (39, 40); therefore, it is likely to impact antibody Fc-effector function. Prior papers use nomenclature that is the reverse of our variant designations: V215I vs I215V and V211M vs M221V. These differences are attributed to the chosen reference sequence for the studies. Interestingly, our data demonstrated that a higher percentage of monkeys sequenced carried the I215 and M211 variants of FcγRIII. This indicates that the reference FcγRIII allele does not actually reflect the major allele most commonly observed in our sample population.

### 3.3 FcγR short structural variants

We next assessed FcγR short structural variants in FcγRI and FcγRIIa. In FcγRI, we found a frameshift insertion, denoted as V6fs, resulting from a duplication of adenine at position 66. This variant occurred in exon 3 and was found in six of the animals we sequenced (**Table 1**). In contrast, a majority of animals (n=140) exhibited a non-frameshift insertion in the cytoplasmic tail of FcγRIIa (**Figure 3C**), denoted as R264delinsDR. This variant occurred in exon 7 and resulted from a three-nucleotide insertion at position 894 (**Table 2**). We identified no short structural variants in either FcγRIIb or FcγRIII (**Tables 3**, **4**).

### 3.4 FcγR isoforms

**Figure 6:**
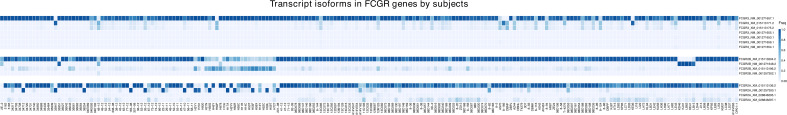
Frequency of expression of FcγR isoforms. The heatmap reports relative abundance of FcγR isoforms identified in 206 Indian-origin rhesus macaques using long-read RNA sequencing. Only isoforms that account for at least 1% of total transcripts in a given FcγR gene in at least one animal are shown. Each row denotes a specific isoform from the FcγR genes FcγRIIa, FcγRIIb or FcγRIII (FcγRI is not represented as it only has one reference isoform). Columns correspond to individual macaques.

**Figure 7:**
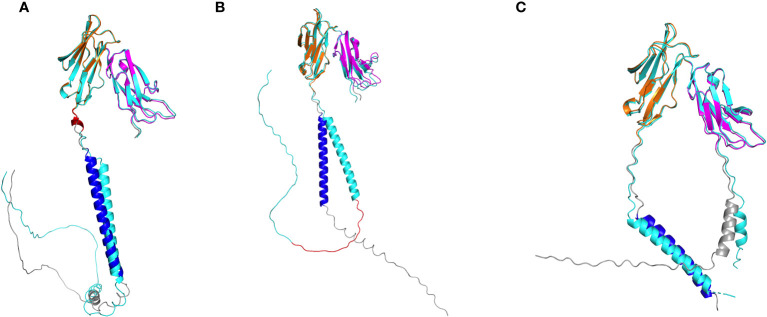
Alphafold2 structural predictions of FcγR isoforms. **(A)** FcγRIIa isoform (cyan) resulting from 5-amino-acid insertion (red) in region between domain 2 and transmebrane domain. **(B)** FcγRIIb isoform (cyan) resulting from 19-amino-acid insertion (red) in cytoplasmic tail. **(C)** FcγRIII isoform (cyan) resulting from single amino acid insertion in signal peptide. In each panel, the reference isoform is colored accordingly: domain 1 (magenta), domain 2 (orange), transmembrane domain (blue), all other regions (gray).

We then evaluated isoforms expressed at a frequency greater than >1% of the total per-gene transcripts in at least one monkey by aligning FcγR reads to established macaque reference isoforms (Rhesus FcγRI has only one reference isoform and was excluded from this analysis) (**Figure 6**; **Table 5**). Here, we adopt the convention of indicating transcript variation relative to the most abundant isoform per FcγR gene in our cohort of monkeys (see isoform sequence alignments, **Figures S3**–**S5**). For FcγRIIa, we detected all four reference isoforms, each distinguished by a unique combination of two variable regions (**Figure S3**). These regions consist of a single amino acid deletion of an alanine at position 36 within the signal peptide and a 5-amino-acid insertion at positions 207-211 in the extracellular portion. The 5-amino-acid insertion corresponds to an alternative 5’ splice site in exon 5 that includes 15 additional nucleotides. The two isoforms with the 5-amino-acid insertion were carried in 111 of 206 macaques (**Table 5**). Interestingly, the five-amino-acid insertion was unique to rhesus macaques; we did not observe the insertion in human FcγRIIa reference isoforms (**Figure S3**). We performed AlphaFold2 structural prediction on an isoform with the 5-amino-acid insertion which revealed that the insertion is located between the transmembrane domain and the immunoglobulin-like domains and is mostly helical (**Figure 7A**). The Alphafold2 structural prediction suggests that the insertion could play a functional role by increasing the distance between the Fc binding regions and the cell surface.

**Table 5:** Frequency of rhesus macaque FcγR isoforms in 206 Indian-origin rhesus macaques using long-read RNA sequencing.

Nucleotide Isoform^1^	Translated Protein Isoform^2^	% Carriers^3^
FCGR2A_XM_015113136.2	XP_014968622.2	93.2 (192/206)
FCGR2A_XM_028846297.1	XP_028702130.1	52.9 (109/206)
FCGR2A_NM_001257300.1	NP_001244229.1	41.7 (86/206)
FCGR2A_XM_028846295.1	XP_028702128.1	30.1 (62/206)
FCGR2B_XM_015113204.2	XP_014968690.1	97.6 (201/206)
FCGR2B_XM_015113196.2	XP_014968682.1	95.1 (196/206)
FCGR2B_NM_001271648.2	NP_001258577.2	4.4 (9/206)
FCGR2B_NM_001257302.1	NP_001244231.1	0.48 (1/206)
FCGR3_NM_001271657.1	NP_001258586.1	97.6 (201/206)
FCGR3_XM_015113171.2	XP_014968657.2	68.4 (141/206)
FCGR3_XM_015113175.2	XP_014968661.2	65.0 (134/206)
FCGR3_NM_001271654.1	NP_001258583.1	1.94 (4/206)
FCGR3_NM_001271653.1	NP_001258582.1	1.46 (3/206)
FCGR3_NM_001271656.1	NP_001258585.1	1.46 (3/206)
FCGR3_NM_001271655.1	NP_001258584.1	0.97 (2/206)

Only isoforms carried in 1% transcripts in at least one animal are shown.

^1^Nucleotide isoform collected from NCBI. ^2^Translated protein isoform collected from NCBI. ^3^percentage of carriers of 206 sequenced animals.

FcγRIIb has four known isoform reference sequences. Two of these isoforms contain a 19-amino-acid insertion in the cytoplasmic domain, starting at position 257, relative to the most abundant FcγRIIb isoform (**Figure S4**). The 19-amino-acid insertion is generated by alternative splicing by including exon 6. While isoforms resulting from exon skipping of exon 6 were found in all 206 sequenced macaques, the isoforms including exon 6 were detected in 196 macaques. In addition, the isoforms including exon 6 had lower relative expression than isoforms without exon 6 in most monkeys (179 out of 196) (**Figure 6**). Alphafold2 structural predictions for both isoforms showed the truncated cytoplasmic tail in the isoform without exon 6 (**Figure 7B**). The exon 6 including rhesus macaque FcγRIIb isoform is similar to the human isoform FcγRII-B1 (NP_003992.3 in **Figure S4**), which also features an almost identical 19-amino-acid insertion in the cytoplasmic tail region, relative to the FcγRII-B2 isoform (NP_001002273.1) (41).

Finally, for FcγRIII, we detected all seven known rhesus macaque isoforms expressed at >1% frequency in at least one monkey in our cohort. These isoforms differ in length only by a single amino acid insertion in the signal peptide (**Figure S5**, **Figure 7C**). The isoforms with the single amino acid insertion were carried by 134 sequenced rhesus macaques, while isoforms without this insertion were found in all 206 macaques (**Figure 6**).

## 4 Discussion

This study designed novel FcγR sequencing primers to the 5′ and 3′ UTR of each FcγR gene allowing for amplification of all rhesus FcγR genes in a large set of rhesus macaques, followed by PacBio long-read sequencing for full-length sequencing of the actively transcribed genes. After sequencing FcγR genes from 206 Indian-origin rhesus macaques, we identified six nonsynonymous SNPs in FcγRI, seven in FcγRIIa, five in FcγRIIb and four in FcγRIII that occur in more than 2% of sequenced animals. Several of the identified SNPs occur in predicted IgG amino acid contact sites (FcγRIIa H131P and N128K, FcγRIIb T119A and S126A, and FcγRIII I158V) and therefore have the potential to impact the response to immune complex formation (27). FcγRIIA M268L and FcγRIII V251I and V221M are located in the cytoplasmic tail, and thus may influence cell signaling. We also identified one SNP (FcγRIIa N128K) that occurs in an N-linked glycosylation site (26), which may alter the receptor function and antibody binding properties (42).

We identified six nonsynonymous SNPs in FcγRI (V28A, R67S, V60A, Q25E, L136F, P12L), and three were previously identified in other studies (V28A, V60A, and R67S) (26, 27). Chan et al. evaluated three allotypes of FcγRI that consisted of the possible combinations of V28A and R67S and found that these allotypes had similar IgG binding affinities. Further, all three variants preferentially bound rhesus IgG1>IgG3>IgG4=IgG2. In contrast, the rhesus FcγRI allotypes bound human IgG3>IgG1>IgG4>IgG2 (27). We identified three additional nonsynonymous SNPs and a frameshift insertion that are not documented in the literature. All identified SNPs occur in the extracellular domain of FcγRI. None of the nonsynonymous SNPs in FcγRI occur in IgG binding regions, so it is unlikely that these SNPs would directly alter IgG binding properties. However, it remains possible that some of these mutations may contribute to protein stability and thus indirectly influence surface expression or antibody interactions.

We identified seven nonsynonymous SNPs in FcγRIIa, and all but M268L were previously described (26, 27, 29). The functional implications for 6 of these have not yet been investigated; the sole exception is H131P SNP, which is analogous to the well characterized human FcγRIIa H/R SNP at position 131 known to impact binding interactions with IgG (29). Recently, Grunst and collaborators used JNL reporter cells transduced to express membrane bound receptors and determined that the rhesus macaque H131 variant had improved IgG binding when compared to the P131 variant (29). Prior work from the Ackerman laboratory evaluated binding activities of four observed allotypes of FcγRIIa using BioLayer Interferometry (BLI), Surface Plasmon Resonance (SPR), and multiplex IgG binding analyses. Allotype FcγR2A-4 was the only one to contain the H131P SNP, and they observed lower binding affinities for this allotype compared to the others (27). Because both Grunst et al. and Chan et al. evaluated H131P in cells expressing specific allotypes, other SNPs were also present which complicates the ability to interpret how individual SNPs may influence functional activity. However, in the study performed by Grunst, the rhesus allotypes expressing H131 all bound more strongly to rhesus IgG2 indicating that H131 likely does contribute to higher binding properties in the presence of the other SNPs (29).

The FcγRIIa and FcγRIIb genes are highly similar in the extracellular regions but have differing transmembrane and cytoplasmic domains (43). We identified five nonsynonymous SNPs in FcγRIIb (N133D, S126A, Q140R, T119A, I125K). Two were previously identified (N133D and I125K) and three also occur in FcγRIIa (N133D, Q140R, I125K) (27). T119A and S126A are documented in the literature as occurring in FcγRIIa (27, 29), but we detected these two SNPs only in FcγRIIb. It is possible that differences in methodologies made it difficult for other studies to assign these SNPs specifically to FcγRIIb. Both T119A and S126A occur in IgG binding domains, and future studies are needed to evaluate their potential impacts on antibody binding interactions. Chan et al. also identified two allotypes of FcγRIIb based on SNPs I14T and L88P. L88P occurs in a predicted IgG binding region, and the allotype containing L88P had very poor binding ability to all rhesus IgGs. The binding defects were also observed when the allotype was evaluated with human IgGs (27). We did not identify either the I14T or L88P in the animals we sequenced. Although the 206 rhesus macaques we sequenced for this project were sourced from multiple studies and colonies, this difference indicates that additional genetic diversity exists beyond what we have described and supports continued research in this area.

We identified four nonsynonymous SNPs in FcγRIII (V125I, V211M, I158V, A8S). Three SNPs were discovered previously (I158V, V215I, and V211M) (27–29). We are the first to identify A8S, which occurs in the extracellular domain. Studies of human FcγRIII genetic diversity identified that the V158 of FcγRIII has higher binding affinity for IgG1 (39). Given that the rhesus FcγRIII SNP occurs at the same location, studies have evaluated the functional impact of rhesus FcγRIII I158V. The recent study by Grunst and collaborators did not identify any differences between FcγRIII allotypes that included I158 and V158 variants in IgG binding capabilities when using Jurkat cells transduced to express FcγRIII allotypes and a NFAT-driven luciferase reporter (JNL cells), including no differences in rhesus IgG subclass binding (29). This study did show that Rhesus FcγRIII allotypes demonstrate a hierarchy in subclass binding to human IgGs: huIgG1>IgG3=IgG4 and minimal responses to huIgG2 (29). Prior work by Chan and collaborators also found that the I158 allotype (FcγR3A-1) had slightly higher binding capabilities than the V158 allotype (FcγR3A-3) when measured using BLI although the differences were not significant (27). The greater IgG binding capabilities of I158 compared to V158 was confirmed by Tolbert et al., except for rhesus FcγRIII V158 binding more tightly to rhesus IgG4 than I158 (38). Further, both Chan et al. and Tolbert et al. observed that FcγRIII IgG binding affinity can be altered based on FcγR glycosylation status (27, 38). Tolbert et al. also evaluated whether the I158 or V158 variant impacts the level of ADCC (29). They demonstrated that NK-92 clones expressing high levels of Rhesus macaque FcγRIII I158 have slightly increased ADCC capabilities when used as effector cells with either the human A32 IgG1 monoclonal antibody or the rhesus macaque J4R monoclonal antibody (38). As differences in glycosylation status have been found to impact the affinity of FcγRIII for IgG, it is possible glycovariation contributed the phenotypic impact observed for the 158I variant in the Tolbert study and the lack of a difference observed in the Grunst study.

Grunst and colleagues also tested allotypes of FcγRIII that included either the V211M or V215I variant in the JNL reporter cell assay and found similar responses in IgG binding across allotypes, suggesting the SNPs in the transmembrane domain and cytoplasmic domain do not have significant effects on signaling in this model. In contrast, an *in vivo* study by Miller et al. found that monkeys with FcγRIII SNPs 211M and 215I (numbered Met229 and Iso233 within reference) were more refractory to CD20 depletion with rituximab when compared to monkeys with the V/V variants (44). These findings suggest that SNPs in FcγRIII transmembrane domain could have *in vivo* importance that cannot be captured *in vitro*. The majority of monkeys we evaluated also express 211M and 215I, and future studies will need to further evaluate the functional implications of these SNPs. Future work should also focus on testing the impact of each SNP on *in vivo* protein expression and conformational presentation on different cell types that may relate to Fc-FcγR function. Future passive antibody administration and vaccination studies that include FcγR sequencing will be critical for providing phenotypic evidence for functionally significant alleles.

In addition to novel SNPs, we detected expression of four isoforms of FcγRIIa, four isoforms of FcγRIIb and seven isoforms of FcγRIII in our study cohort. To identify isoforms within our sequencing data, we aligned our FcγR reads to established macaque reference isoforms. Two rhesus FcγRIIa isoforms have a small insertion (five amino acids) in the extracellular domain, which is predicted to increase the distance between the cell surface and the receptor. These isoforms may impact antibody binding interactions and future studies will evaluate the functional impact of this insertion. A single amino acid deletion also observed in human isoforms is present in two of the rhesus isoforms (**Figure S3**). In our cohort, we detected macaques expressing two previously identified FcγRIIb isoforms, each with a 19 amino acid insertion in the cytoplasmic domain, which may modify signal transduction. An isoform with an almost identical 19 amino acid insertion also exists in humans (FcγRII-B1) (41). This insertion may have a role in inhibition of endocytosis resulting in prolonged surface expression levels (45–47). In mice, FcγRIIb isoforms are differentially expressed based on cell types. This isoform is found at slightly higher levels in inflammatory macrophages compared to homeostatic macrophages in humans (48), but future studies to determine cell type expression levels in rhesus macaques are needed. Two of the rhesus FcγRIIb isoforms have a single amino acid insertion, which is also found in the human isoform FcγRII-B2 (**Figure S4**). The FcγRIII isoforms differ by the insertion of one amino acid in the signal peptide sequence (**Figure S5**). Future studies are needed to further elucidate the functional impact of all rhesus FcγR isoforms.

Using long-read RNA sequencing, we characterized the genetic diversity of 206 Indian-origin rhesus macaque FcγR. We confirmed three nonsynonymous SNPs in FcγRI, six in FcγRIIa, two in FcγRIIb, and three in FcγRIII that were already described (26–29). We also discovered 8 novel nonsynonymous SNPs: three in FcγRI, one in FcγRIIa, three in FcγRIIb, and one in FcγRIII. Long read sequencing also facilitated identification of transcript structural variation, which allowed us to characterize isoforms among our cohort. We identified four isoforms in FcγRIIa, four in FcγRIIb, and seven in FcγRIII in our study cohort. Our investigation demonstrates that the rhesus macaque FcγR genome is more complex than currently appreciated. Moreoever, while 206 rhesus macaques is a large cohort of animals, we do not rule out that future FcγR sequencing may discover even more diversity of SNPs and isoforms.

Other macaque species, such as cynomolgus macaques (*Macaca fascicularis*) and pig-tailed macaques (*Macaa nemestrina*) are also frequently used for vaccination and passive antibody administration experiments. FcγR information for these macaque species is also very limited. Sequencing analysis of cynomolgus macaques show one SNP in FcγRI (Q25E) similar to rhesus macaques in our study (27). There are similarities in FcγRIIa and FcγRIIb with rhesus, cynomolgus, and pig-tailed macaques sharing many SNPs (27, 28, 49, 50). Both cynomolgus and rhesus macaques have the FcγRIII I158V SNP in the IgG contact region (27, 28). Based on our investigation of rhesus macaques, cynomolgus and pig-tailed macaques may also have greater FcγR genetic diversity than what is currently known.

Our study expands on previous work to further characterize the genetic diversity of Indian-origin rhesus macaques. The few *in vitro* studies performed have shown that some models of IgG binding are impacted by FcγR SNPs; however, more *in vivo* and *in vitro* studies are needed to further elucidate the impact of FcγR genetic diversity on clinical outcomes. The functionality of individual SNPs *in vivo* may also be influenced by homozygosity/heterozygosity, FcγR gene copy number, activation levels of FcγR-bearing cells, linkage disequilibrium, etc.; therefore, future studies should consider all these factors in when determining the functional role of SNPs. Rhesus macaque FcγR genetic diversity has the potential to confound study outcomes, thus including FcγR sequencing to quantify this effect in humoral immunity-based studies is critical.

## Data availability statement

The data presented in the study are deposited in The National Center for Biotechnology (NCBI) BioProject Repository, accession number PRJNA1030012 (https://www.ncbi.nlm.nih.gov/bioproject).

## Ethics statement

Leftover blood samples from completed studies at multiple institutions were used for the current study. These blood samples represent 206 Indian origin rhesus macaques, sourced from several different vendors, providers, or colonies. All blood was collected in accordance with protocols approved by the appropriate Institutional Animal Care and Use Committee. The study was conducted in accordance with the local legislation and institutional requirements.

## Author contributions

HC: Investigation, Methodology, Writing – original draft, Writing – review & editing. MH: Data curation, Formal analysis, Writing – review & editing. DE: Investigation, Methodology, Writing – original draft, Writing – review & editing. HK: Writing – review & editing, Formal analysis, Methodology, Visualization. SC: Writing – review & editing, Conceptualization. JM: Investigation, Writing – review & editing. TH: Investigation, Writing – review & editing. MB: Writing – review & editing, Investigation. TB: Writing – review & editing, Investigation. WT: Writing – review & editing, Formal analysis, Visualization. MP: Writing – review & editing, Formal analysis, Visualization. GT: Funding acquisition, Writing – review & editing, Supervision. JS: Writing – review & editing, Conceptualization. MM: Supervision, Writing – review & editing, Conceptualization. KW: Methodology, Supervision, Writing – review & editing, Formal analysis, Visualization. JP: Supervision, Writing – original draft, Writing – review & editing, Formal analysis, Conceptualization, Funding acquisition.
