# Supplementary material for: Defining genetic diversity of rhesus macaque Fcγ receptors with long-read RNA sequencing
Source: Front Immunol. 2024 Jan 9;14:1306292. doi: 10.3389/fimmu.2023.1306292 (PMC10803544; doi:10.3389/fimmu.2023.1306292)
Supplement: Supplementary file 1 [file DataSheet_1.pdf]

Supplementary Material

Defining genetic diversity of rhesus macaque Fcγ receptors with long-read RNA sequencing

Haleigh E. Conley[1], Max M. He[2], David Easterhoff[2#], Hélène Fradin Kirshner[2], Sarah L. Cocklin[3], Jacob Meyer[2], Taylor Hoxie[2], Madison Berry[2], Todd Bradley[4], William D. Tolbert[5], Marzena Pazgier[5], Georgia D. Tomaras[1,2], Joern E. Schmitz[3], M. Anthony Moody[2]†, Kevin Wiehe[2]†*, Justin Pollara[1]†*

*** Correspondence:** Justin Pollara, justin.pollara@duke.edu; Kevin Wiehe, kevin.wiehe@duke.edu

A.

Species	Gene	Primer Direction	Sequence (5' to 3')
macaca mulatta	FCGR1A	Forward	Barcode - CATGTTACAGATTTCGCTGCTCC
macaca mulatta	FCGR1A	Reverse	Barcode – CACCCACTGAGCTGCTGCTA
macaca mulatta	FCGR2A	Forward	Barcode – GACTGGACGTTGGCACAGTGCTGG
macaca mulatta	FCGR2A	Reverse	Barcode – CCACTCAGCAAGCTGAGAGTGTGACCAC
macaca mulatta	FCGR2B	Forward	Barcode – CAAACTTGGAGAGAAGGCTGTGACTG
macaca mulatta	FCGR2B	Reverse	Barcode – CTCAAATCCCAAGGCAAGACAATGGAG
macaca mulatta	FCGR3	Forward	Barcode – GATGCTGCTGCCACTGCTCTTATTAC
macaca mulatta	FCGR3	Forward	Barcode – CTCCCAGTCCCTTGTTGAGCTTG
macaca mulatta	FCGR3	Reverse	Barcode – GCCAGCCTTCAGATTGAGAAGTCAG

B.

Thermal Cycler Conditions		
94˚C	2 minutes	x 1 cycle
94˚C	30 seconds	
64˚C	30 seconds	x 34 cycles
72˚C	1 minute	
72˚C	7 minutes	x 1 cycle
4˚C	Infinite hold	

C.

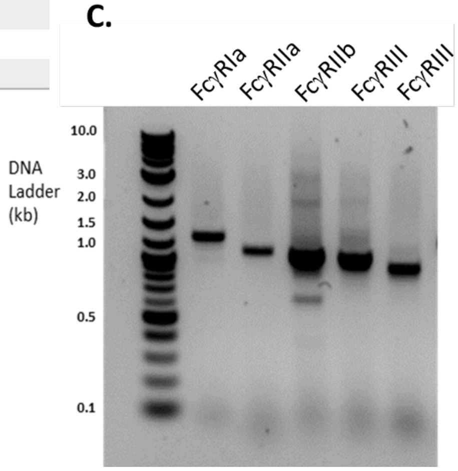

Supplementary Figure 1. Rhesus macaque FcγR type I gene specific primers. (A) PCR primers were designed to the 5' and 3' untranslated region of FcγRIa, FcγRIIa, FcγRIIb and FcγRIII. Multiple FcγRIII sequences with different 5' regions were identified so to increase primer coverage two different FcγRIII forward primers were used. (B) PCR thermocycler conditions using Invitrogen Platinum (II) Hot Start PCR Master Mix. (C) PCR amplicons were visualized on a 1% agarose gel with the 1kb Plus DNA ladder (NEB). PCR amplicons are of the expected molecular weight.

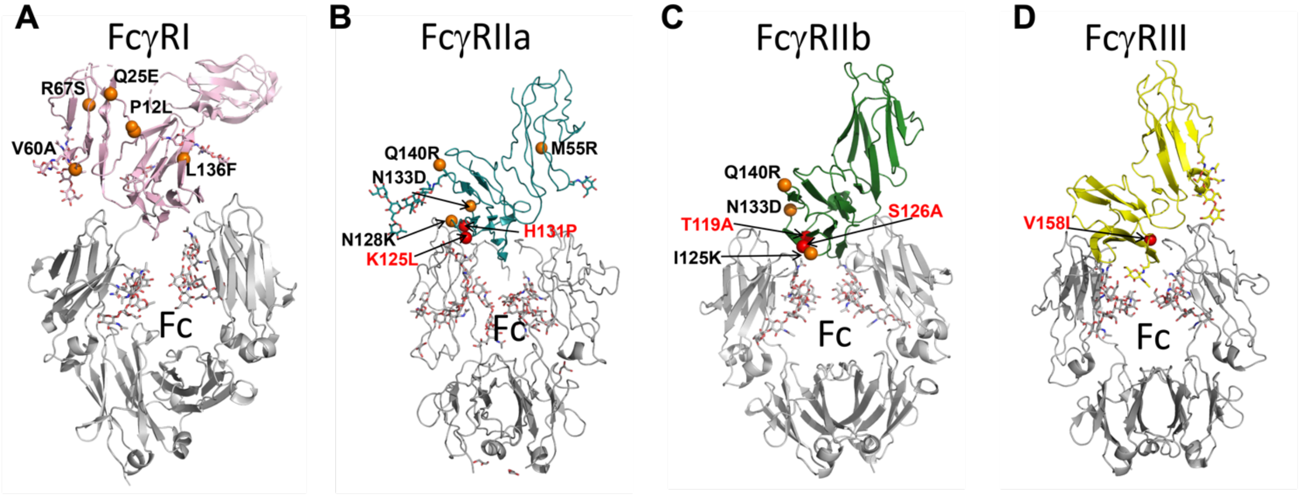

Supplemental Figure 2. Predicted interactions of Rhesus macaque FcγR interactions with antibody Fc region. Observed SNPs are shown. SNPs shown in red occur at the IgG binding interface. (A) FcγRI – no SNPs at binding interface, (B) FcγRIIa – SNPs H131P, K125I at binding interface, (C) FcγRIIb – SNPs T119A, S126A at binding interface, (D) FcγRIII – I158V at binding interface.

```
                                        10        20        30        40        50        60        70        80        90       100
                                    :    |    :    |    :    |    :    |    :    |    :    |    :    |    :    |    :    |    :    |
XP_014968622.2 [Macaca mulatta] MTMETQMSQNVCPSNLWLLQPLTVLLLLASADSQTAAPPKAVLKLEPPWINVLREDSVTLTCGGAHSPDSDSTQWFHNGNLIPTHTQPSYMFKANNNDSG
XP_028702130.1 [Macaca mulatta] ...................................-................................................................
NP_001244229.1 [Macaca mulatta] ...................................-................................................................
XP_028702128.1 [Macaca mulatta] ....................................................................................................
NP_001129691.1 [Homo sapiens]   .............R....................A..................Q........Q..R..E...I.................R.........
NP_067674.2    [Homo sapiens]   .............R....................-..................Q........Q..R..E...I.................R.........
NP_001362225.1 [Homo sapiens]   .............R....................A..................Q........Q..R..E...I.................R.........
NP_001362226.1 [Homo sapiens]   .............R....................-..................Q........Q..R..E...I.................R.........
XP_011507592.1 [Homo sapiens]   .............R....................A..................Q........Q..R..E...I.................R.........
XP_011507593.1 [Homo sapiens]   .............R....................A..................Q........Q..R..E...I.................R.........
XP_016856152.1 [Homo sapiens]   .............R....................-..................Q........Q..R..E...I.................R.........
XP_016856153.1 [Homo sapiens]   .............R....................A..................Q........Q..R..E...I.................R.........
XP_016856154.1 [Homo sapiens]   .............R....................A..................Q........Q..R..E...I.................R.........
XP_016856155.1 [Homo sapiens]   .............R....................A..................Q........Q..R..E...I.................R.........
XP_024309808.1 [Homo sapiens]   ----------------------------------------------------------------------------------------------------
XP_047305397.1 [Homo sapiens]   .............R....................A..................Q........Q..R..E...I.................R.........
XP_054191107.1 [Homo sapiens]   .............R....................A..................Q........Q..R..E...I.................R.........
XP_054191108.1 [Homo sapiens]   .............R....................-..................Q........Q..R..E...I.................R.........
XP_054191109.1 [Homo sapiens]   .............R....................A..................Q........Q..R..E...I.................R.........
XP_054191110.1 [Homo sapiens]   .............R....................A..................Q........Q..R..E...I.................R.........
XP_054191111.1 [Homo sapiens]   .............R....................A..................Q........Q..R..E...I.................R.........
XP_054191112.1 [Homo sapiens]   .............R....................A..................Q........Q..R..E...I.................R.........
XP_054191113.1 [Homo sapiens]   ----------------------------------------------------------------------------------------------------

                                       110       120       130       140       150       160       170       180       190       200
                                    :    |    :    |    :    |    :    |    :    |    :    |    :    |    :    |    :    |    :    |
XP_014968622.2 [Macaca mulatta] EYRCQTGRTSLSDPVHLTVLSEWLALQTTHLEFREGETIMLRCHSWKDKPLIKVAFFQNGKSKNFSHMNPNFSIPQANHSHSGDYHCTGNIGYTPYSSKP
XP_028702130.1 [Macaca mulatta] ....................................................................................................
NP_001244229.1 [Macaca mulatta] ....................................................................................................
XP_028702128.1 [Macaca mulatta] ....................................................................................................
NP_001129691.1 [Homo sapiens]   ..T....Q................V...P....Q.................V..T.......QK...LD.T.......................LF....
NP_067674.2    [Homo sapiens]   ..T....Q................V...P....Q.................V..T.......QK...LD.T.......................LF....
NP_001362225.1 [Homo sapiens]   ..T....Q................V...P....Q.................V..T.......QK...LD.T.......................LF....
NP_001362226.1 [Homo sapiens]   ..T....Q................V...P....Q.................V..T.......QK...LD.T.......................LF....
XP_011507592.1 [Homo sapiens]   ..T....Q................V...P....Q.................V..T.......QK...LD.T.......................LF....
XP_011507593.1 [Homo sapiens]   ..T....Q................V...P....Q.................V..T.......QK...LD.T.......................LF....
XP_016856152.1 [Homo sapiens]   ..T....Q................V...P....Q.................V..T.......QK...LD.T.......................LF....
XP_016856153.1 [Homo sapiens]   ..T....Q................V...P....Q.................V..T.......QK...LD.T.......................LF....
XP_016856154.1 [Homo sapiens]   ..T....Q................V...P....Q.................V..T.......QK...LD.T.......................LF....
XP_016856155.1 [Homo sapiens]   ..T....Q................V...P....Q.................V..T.......QK...LD.T.......................LF....
XP_024309808.1 [Homo sapiens]   ---------------------------------------............V..T.......QK...LD.T.......................LF....
XP_047305397.1 [Homo sapiens]   ..T....Q................V...P....Q.................V..T.......QK...LD.T.......................LF....
XP_054191107.1 [Homo sapiens]   ..T....Q................V...P....Q.................V..T.......QK..RLD.T.......................LF....
XP_054191108.1 [Homo sapiens]   ..T....Q................V...P....Q.................V..T.......QK..RLD.T.......................LF....
XP_054191109.1 [Homo sapiens]   ..T....Q................V...P....Q.................V..T.......QK..RLD.T.......................LF....
XP_054191110.1 [Homo sapiens]   ..T....Q................V...P....Q.................V..T.......QK..RLD.T.......................LF....
XP_054191111.1 [Homo sapiens]   ..T....Q................V...P....Q.................V..T.......QK..RLD.T.......................LF....
XP_054191112.1 [Homo sapiens]   ..T....Q................V...P....Q.................V..T.......QK..RLD.T.......................LF....
XP_054191113.1 [Homo sapiens]   ---------------------------------------............V..T.......QK..RLD.T.......................LF....

                                       210       220       230       240       250       260       270       280       290       300
                                    :    |    :    |    :    |    :    |    :    |    :    |    :    |    :    |    :    |    :    |
XP_014968622.2 [Macaca mulatta] VTITVQ-----VPSVGSSSPMGIIVAVVTGIAVVAIVAAVVALIYCRKKRISANSTDPVKAARNEPLGRQTIALRKRQLEETNND---YETADGGYMTLN
XP_028702130.1 [Macaca mulatta] ......GLSSL.........................................................................................
NP_001244229.1 [Macaca mulatta] ....................................................................................................
XP_028702128.1 [Macaca mulatta] ......GLSSL.........................................................................................
NP_001129691.1 [Homo sapiens]   ..............M.............IAT..A............................QF..P...M..I..........................
NP_067674.2    [Homo sapiens]   ..............M.............IAT..A............................QF..P...M..I..........................
NP_001362225.1 [Homo sapiens]   ...........-----------------------------------------..........QF..P...M..I..........................
NP_001362226.1 [Homo sapiens]   ...........-----------------------------------------..........QF..P...M..I..........................
XP_011507592.1 [Homo sapiens]   ..............M.............IAT..A............................QF..P...M..I..........................
XP_011507593.1 [Homo sapiens]   ..............M.............IAT..A...................TW.S--------------------------..CHQK..T--------
XP_016856152.1 [Homo sapiens]   ..............M.............IAT..A............................QF..P...M..I..........................
XP_016856153.1 [Homo sapiens]   ..............M.............IAT..A............................QF..P...M..I..........................
XP_016856154.1 [Homo sapiens]   ..............M.............IAT..A............................QF..P...M..I..........................
XP_016856155.1 [Homo sapiens]   ...........-----------------------------------------..........QF..P...M..I..........................
XP_024309808.1 [Homo sapiens]   ..............M.............IAT..A............................QF..P...M..I..........................
XP_047305397.1 [Homo sapiens]   ..............M.............IAT..A............................QF..P...M..I..........................
XP_054191107.1 [Homo sapiens]   ..............M.............IAT..A............................QF..P...M..I..........................
XP_054191108.1 [Homo sapiens]   ..............M.............IAT..A............................QF..P...M..I..........................
XP_054191109.1 [Homo sapiens]   ..............M.............IAT..A............................QF..P...M..I..........................
XP_054191110.1 [Homo sapiens]   ..............M.............IAT..A............................QF..P...M..I..........................
XP_054191111.1 [Homo sapiens]   ...........-----------------------------------------..........QF..P...M..I..........................
XP_054191112.1 [Homo sapiens]   ..............M.............IAT..A............................QF..P...M..I..........................
XP_054191113.1 [Homo sapiens]   ..............M.............IAT..A............................QF..P...M..I..........................

                                       310       320
                                    :    |    :    |    :
XP_014968622.2 [Macaca mulatta] PRAPT-DDRNIYMTLSPNDYDNSNN
XP_028702130.1 [Macaca mulatta] .........................
NP_001244229.1 [Macaca mulatta] .........................
XP_028702128.1 [Macaca mulatta] .........................
NP_001129691.1 [Homo sapiens]   .....D..K...L..P...HV....
NP_067674.2    [Homo sapiens]   .....D..K...L..P...HV....
NP_001362225.1 [Homo sapiens]   .....D..K...L..P...HV....
NP_001362226.1 [Homo sapiens]   .....D..K...L..P...HV....
XP_011507592.1 [Homo sapiens]   .....D..K...L..P...HV.RLY
XP_011507593.1 [Homo sapiens]   -----.-------------------
XP_016856152.1 [Homo sapiens]   .....D..K...L..P...HV.T.K
XP_016856153.1 [Homo sapiens]   .....D..K...L..P...HV.TEI
XP_016856154.1 [Homo sapiens]   .....D..K...L..P...HV.TEI
XP_016856155.1 [Homo sapiens]   .....D..K...L..P...HV.T.K
XP_024309808.1 [Homo sapiens]   .....D..K...L..P...HV.T.K
XP_047305397.1 [Homo sapiens]   .....D..K...L..P...HV.T.K
XP_054191107.1 [Homo sapiens]   .....D..K...L..P...HV.T.K
XP_054191108.1 [Homo sapiens]   .....D..K...L..P...HV.T.K
XP_054191109.1 [Homo sapiens]   .....D..K...L..P...HV.TEI
XP_054191110.1 [Homo sapiens]   .....D..K...L..P...HV.TEI
XP_054191111.1 [Homo sapiens]   .....D..K...L..P...HV.T.K
XP_054191112.1 [Homo sapiens]   .....D..K...L..P...HV.RLY
XP_054191113.1 [Homo sapiens]   .....D..K...L..P...HV.T.K
```

Supplemental Figure 3. Rhesus macaque FcγRIIa isoform alignment with human FcγRIIa reference isoforms.

```
                                        10        20        30        40        50        60        70        80        90       100
                                    :    |    :    |    :    |    :    |    :    |    :    |    :    |    :    |    :    |    :    |
XP_014968690.1 [Macaca mulatta] MGILSFLPVLATESDWADCKSSQPWGHMLLWTAVLFLAPVAGTP-APPKAVLKLEPPWINVLREDSVTLTCGGAHSPDSDSTQWFHNGNLIPTHTQPSYR
XP_014968682.1 [Macaca mulatta] ....................................................................................................
NP_001258577.2 [Macaca mulatta] ............................................A.......................................................
NP_001244231.1 [Macaca mulatta] ............................................A.......................................................
NP_001002273.1 [Homo sapiens]   .....................P..................................Q.....Q........R.T...E...I..................
NP_003992.3    [Homo sapiens]   .....................P......................A...........Q.....Q........R.T...E...I..................

                                       110       120       130       140       150       160       170       180       190       200
                                    :    |    :    |    :    |    :    |    :    |    :    |    :    |    :    |    :    |    :    |
XP_014968690.1 [Macaca mulatta] FKANNNDSGEYRCQTGRTSLSDPVHLTVLSEWLALQTPHLEFREGETIMLRCHSWKDKPLIKVTFFQNGISKKFSHMNPNFSIPQANHSHSGDYHCTGNI
XP_014968682.1 [Macaca mulatta] ....................................................................................................
NP_001258577.2 [Macaca mulatta] ....................................................................................................
NP_001244231.1 [Macaca mulatta] ....................................................................................................
NP_001002273.1 [Homo sapiens]   ...........T....Q................V........Q.....V...........V........K.....RSD......................
NP_003992.3    [Homo sapiens]   ...........T....Q................V........Q.....V...........V........K.....RSD......................

                                       210       220       230       240       250       260       270       280       290       300
                                    :    |    :    |    :    |    :    |    :    |    :    |    :    |    :    |    :    |    :    |
XP_014968690.1 [Macaca mulatta] GYTPYSSKPVTITVQVPSMGSSSPIGIIVAVVTGIAVAAIVAAVVALIYCRKKRIS-------------------ANPTNPDEADKVGAENTITYSLLMH
XP_014968682.1 [Macaca mulatta] ........................................................ALPGNPECREMGETLPEKP.........................
NP_001258577.2 [Macaca mulatta] ....................................................................................................
NP_001244231.1 [Macaca mulatta] ........................................................ALPGNPECREMGETLPEKP.........................
NP_001002273.1 [Homo sapiens]   ...L...........A.---....M...........................................................................
NP_003992.3    [Homo sapiens]   ...L...........A.---....M...............................ALPGYPECREMGETLPEKP.........................

                                       310
                                    :    |
XP_014968690.1 [Macaca mulatta] PDALEEPDDQNRV
XP_014968682.1 [Macaca mulatta] .............   6.1
NP_001258577.2 [Macaca mulatta] .............   0.3
NP_001244231.1 [Macaca mulatta] .............   6.4
NP_001002273.1 [Homo sapiens]   ............I   7.7
NP_003992.3    [Homo sapiens]   ............I   14.1
```

Supplemental Figure 4. Rhesus macaque FcγRIIb isoform alignment with human FcγRIIb isoforms.

```
                                        10        20        30        40        50        60        70        80        90       100
                                    :    |    :    |    :    |    :    |    :    |    :    |    :    |    :    |    :    |    :    |
NP_001258586.1 [Macaca mulatta] MWQLLLPTALLLLVSAGMRA-DLPKAVVFLEPQWYRVLEKDSVTLKCQGAYSPEDNSTRWFHNESLISSQTSSYFIAAARVNNSGEYRCQTSLSTLSDPV
XP_014968657.2 [Macaca mulatta] ....................................................................................................
XP_014968661.2 [Macaca mulatta] ....................E...............................................................................
NP_001258583.1 [Macaca mulatta] ....................................................................................................
NP_001258582.1 [Macaca mulatta] ....................E...............................................................................
NP_001258585.1 [Macaca mulatta] ....................E...............................................................................
NP_001258584.1 [Macaca mulatta] ....................E...............................................................................
NP_000560.7    [Homo sapiens]   ...................TE.....................................Q...........A.....D..T.DD........N........
NP_001121064.2 [Homo sapiens]   ...................T......................................Q...........A.....D..T.DD........N........

                                       110       120       130       140       150       160       170       180       190       200
                                    :    |    :    |    :    |    :    |    :    |    :    |    :    |    :    |    :    |    :    |
NP_001258586.1 [Macaca mulatta] QLEVHIGWLLLQAPRWVFKEEESIHLRCHSWKNTLLHKVTYLQNGKGRKYFHQNSDFYIPKATLKDSGSYFCRGLIGSKNVSSETVNITITQDLAVSSIS
XP_014968657.2 [Macaca mulatta] ....................................................................................................
XP_014968661.2 [Macaca mulatta] ....................................................................................................
NP_001258583.1 [Macaca mulatta] ....................................................................................................
NP_001258582.1 [Macaca mulatta] ....................................................................................................
NP_001258585.1 [Macaca mulatta] ....................................................................................................
NP_001258584.1 [Macaca mulatta] ....................................................................................................
NP_000560.7    [Homo sapiens]   .....................DP...........A.................H......................F................G....T..
NP_001121064.2 [Homo sapiens]   .....................DP...........A.................H......................F................G....T..

                                       210       220       230       240       250
                                    :    |    :    |    :    |    :    |    :    |
NP_001258586.1 [Macaca mulatta] SFFPPGYQVSFCLVMVLLFAVDTGLYFSMKKSIPSSTRDWEDHKFKWSKDPQDK
XP_014968657.2 [Macaca mulatta] ............................V...V.....................  0.8
XP_014968661.2 [Macaca mulatta] ............................V...V.....................  1.2
NP_001258583.1 [Macaca mulatta] ......................................................  0
NP_001258582.1 [Macaca mulatta] ......................................................  0.4
NP_001258585.1 [Macaca mulatta] ......................................................  0.4
NP_001258584.1 [Macaca mulatta] ......................................................  0.4
NP_000560.7    [Homo sapiens]   ............................V.TN.R......K......R......  8.7
NP_001121064.2 [Homo sapiens]   ............................V.TN.R......K......R......  8.3
```

Supplemental Figure 5. Rhesus macaque FcγRIII isoform alignment with human FcγRIII isoforms.

**Gene**	**FcγRIIA**			**FcγRIII**			
SNP	C855T/ G250G	894_895insGAT/ R264delinsDR	A907C/ M268L	G528A/ T122T	A634G/ I158V	G793A/ V211M	G805A/ V215I
**AR87**							
PacBio	0	0	0	0	1	1	1
Sanger	C	-	A	G	A/G	G/A	G/A
**AS42**							
PacBio	0	0	0	0	1	1	1
Sanger	C	-	A	G	A/G	G/A	G/A
**34720**							
PacBio		1	1	0	0	1	1
Sanger	C	GAT/0	A/C	G	A	G/A	G/A
**53-12**							
PacBio	0	0	0	0	0	0	0
Sanger	C	-	A	G	A	G	G
**36064**							
PacBio	0	2	2	0	0	1	1
Sanger	C	GAT	C	G	A	G/A	G/A
**08D165**							
PacBio	0	0	0	0	0	1	1
Sanger	C	-	A	G	A	G/A	G/A
**8-2**							
PacBio	0	1	0	0	0	2	2
Sanger	C	GAT/0	A	G	A	A	A
**ZE24**							
PacBio	0	0	0	0	0	0	0
Sanger	C	-	A	G	A	G	G
**08D106**							
PacBio	0	1	0	0	1	0	0
Sanger	C	GAT/0	A	G	A/G	G	G
**35464**							
PacBio	0	1	1	0	0	0	0
Sanger	C	GAT/0	A/C	G	A	G	G

Supplemental Table 1. Comparison of PacBio and Sanger Sequencing approaches to detect SNPs. PacBio results: 0 is no detection of SNP, 1 is heterozygous detection of SNP, 2 is homozygous detection of SNP.
